# Study design and rationale of "Synergistic Effect of Combination Therapy with Cilostazol and ProbUcol on Plaque Stabilization and Lesion REgression (SECURE)" study: a double-blind randomised controlled multicenter clinical trial

**DOI:** 10.1186/1745-6215-12-10

**Published:** 2011-01-12

**Authors:** Young-Guk Ko, Byeong-Keuk Kim, Byoung Kwon Lee, Woong Chol Kang, Seung Hyuk Choi, Sang Wook Kim, Jong Ho Lee, Myoungsook Lee, Yasuhiro Honda, Peter J Fitzerald, Won-Heum Shim

**Affiliations:** 1 Severance Cardiovascular Hospital, Yonsei University Health System, Seoul, Korea; 2 Gangnam Severance Hospital, Yonsei University Health System, Seoul, Korea; 3 Gil Medical Center, Gachon University, Incheon, Korea; 4 Samsung Medical Center, Sungkyunkwan University, Seoul, Korea; 5 Chung-Ang University Hospital, Seoul, Korea; 6 National Research Laboratory for Clinical Nutrigenetics/Nutrigenomics, Yonsei University, Seoul, Korea; 7 Laboratory of Nutritional Medicine/Nutrigenomics, Sungshin Women's University, Seoul, Korea; 8 Cardiac Core Analysis Laboratory at Stanford University Medical Center, Stanford, CA, USA

## Abstract

**Background:**

Probucol, a cholesterol-lowering agent that paradoxically also lowers high-density lipoprotein cholesterol has been shown to prevent progression of atherosclerosis. The antiplatelet agent cilostazol, which has diverse antiatherogenic properties, has also been shown to reduce restenosis in previous clinical trials. Recent experimental studies have suggested potential synergy between probucol and cilostazol in preventing atherosclerosis, possibly by suppressing inflammatory reactions and promoting cholesterol efflux.

**Methods/design:**

The Synergistic Effect of combination therapy with Cilostazol and probUcol on plaque stabilization and lesion REgression (SECURE) study is designed as a double-blind, randomised, controlled, multicenter clinical trial to investigate the effect of cilostazol and probucol combination therapy on plaque volume and composition in comparison with cilostazol monotherapy using intravascular ultrasound and Virtual Histology. The primary end point is the change in the plaque volume of index intermediate lesions between baseline and 9-month follow-up. Secondary endpoints include change in plaque composition, neointimal growth after implantation of stents at percutaneous coronary intervention target lesions, and serum levels of lipid components and biomarkers related to atherosclerosis and inflammation. A total of 118 patients will be included in the study.

**Discussion:**

The SECURE study will deliver important information on the effects of combination therapy on lipid composition and biomarkers related to atherosclerosis, thereby providing insight into the mechanisms underlying the prevention of atherosclerosis progression by cilostazol and probucol.

**Trial registration number:**

ClinicalTrials (NCT): NCT01031667

## Background

The benefit of reducing low-density lipoprotein (LDL) cholesterol in the management of coronary artery disease (CAD) has been demonstrated in various large-scale randomized clinical trials [1]. These trials have shown that statin therapy, which is currently considered an essential element in CAD treatment regimens, does improve clinical outcomes, but is only able to prevent coronary events in less than 30% of patients. Therefore, a need remains for new and more effective cardiovascular drug therapies.

Probucol, a mild cholesterol-lowering agent with antioxidant and anti-inflammatory properties, has been shown to reduce atherosclerosis and prevent restenosis after percutaneous coronary intervention (PCI) [2]. However, probucol also causes a remarkable reduction in high density lipoprotein (HDL) and prolongation of QT intervals on electrocardiograms, side effects that have led to the withdrawal of probucol from the market in many countries. Although the mechanisms underlying the antiatherosclerotic effect of probucol remain unclear, previous speculation has centered on inhibition of oxidative modification of LDL cholesterol [3]. However, recent studies have suggested that enhanced reverse cholesterol transport caused by activation of cholesteryl ester transfer protein (CETP) and scavenger reverse cholesterol class B type 1 (SR-B1) is the major mechanism responsible for both the antiatherogenic effect and reduction of HDL levels by probucol [4-6]. The apparent lowering of HDL levels by probucol may be related to a change in the composition of HDL subtypes, including an increase in preβ1-HDL ("lipid-poor" apoA-1), that participate in the cholesterol efflux [7]. Therefore, the lowering of HDL by probucol is not a side effect, per se, but instead may reflect its main effect of increasing cholesterol efflux. Furthermore, probucol has been shown to inhibit mononuclear cell adhesion and reduce expression of vascular cell adhesion molecule (VCAM) [8]. Probucol also promotes nitric oxide-mediated vasodilatation and re-endothelialization, and inhibits smooth muscle cell proliferation [9].

Cilostazol, an inhibitor of type 3 phosphodiesterase that exerts antiplatelet activity through suppression of cyclic adenosine monophosphate degradation [10], also exhibits diverse antiatherogenic properties. Studies have shown that cilostazol improves endothelial function by increasing nitric oxide production [11], promotes scavenging of free radicals [12], and inhibits foam cell formation [13] and smooth muscle cell proliferation [14]. In addition, cilostazol has been shown to decrease triglyceride concentrations and increase HDL cholesterol concentration, possibly by increasing the activity of lipoprotein lipase [15,16].

A previous study by Sekiya et al. [17] reported that cilostazol and probucol combination therapy reduced restenosis after stent implantation more effectively than did either cilostazol or probucol alone. In addition, recent studies have demonstrated potential synergistic effects of cilostazol and probucol used in combination in the prevention of atherosclerosis [18,19]. Yoshikawa et al. [18] found that combined administration of cilostazol and probucol induced a greater decrease in the atherosclerotic lesion area in LDL receptor-deficient mice than did administration of each drug separately. Park et al. [19] showed that cilostazol and probucol combination therapy inhibited the expression of VCAM-1 and monocyte chemoattractant protein-1 (MCP-1) more effectively than did either cilostazol or probucol monotherapy.

Intravascular ultrasound (IVUS) is a useful imaging modality for assessment of atherosclerotic plaque and vessel wall morphology. Especially, Virtual histology (VH) IVUS (Volcano Therapeutics, Inc., Rancho Cordova, California) using spectral analysis of the radiofrequency ultrasound back-scatter signals allows identification and relative quantification of four different components of atherosclerotic plaques: fibrous, fibro-fatty, dense calcium and necrotic core [20-25]. Therefore, the SECURE study is designed to investigate effects of cilostazol and probucol combination therapy on coronary plaque volume and composition using VH-IVUS in comparison with cilostazol monotherapy.

## Methods/design

### Study Design

**Figure 1:**
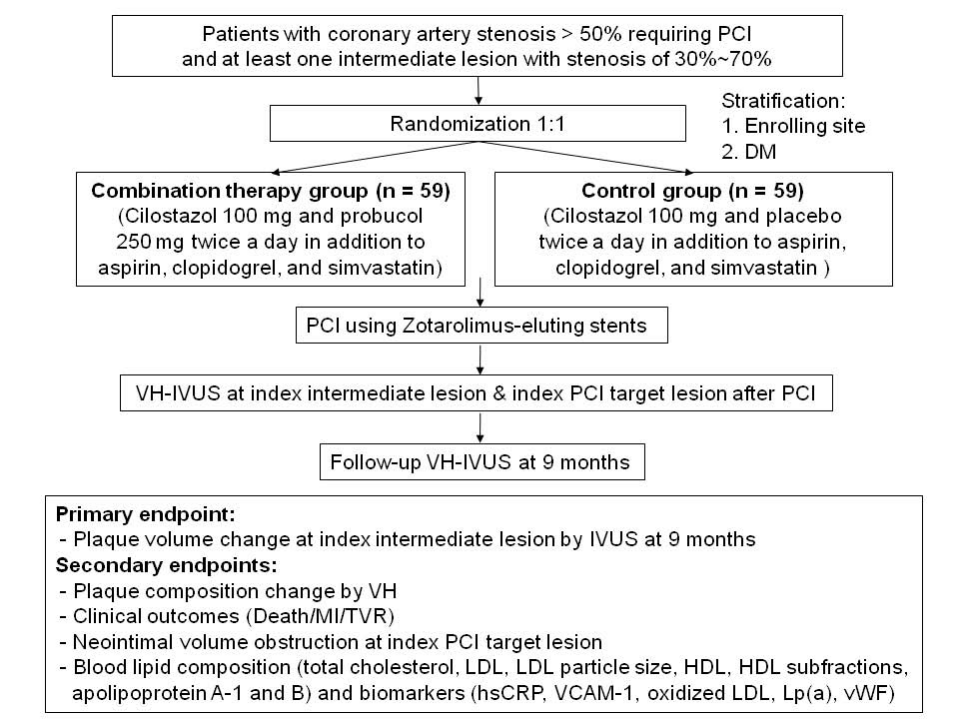
**SECURE study algorithm**. PCI, percutaneous coronary intervention; DM, diabetes mellitus; VH-IVUS, Virtual Histology intravascular ultrasound; MI, myocardial infarction; TVR, target vessel revascularization; LDL, low-density lipoprotein; HDL, high-density lipoprotein; hsCRP, high-sensitivity C-reactive protein; VCAM-1, vascular cell adhesion molecule-1; Lp(a), lipoprotein (a); vWF, von Willebrand factor.

This is a double-blind, placebo-controlled, randomised, multicenter clinical trial to evaluate the efficacy of probucol and cilostazol combination therapy compared with cilostazol alone in the prevention of coronary plaque progression (Figure 1). A total of 118 patients (see Sample size calculation, below) will be enrolled at five PCI centers in Korea. After enrollment and baseline investigation with IVUS and VH to establish index non-PCI target intermediate lesions and stented PCI target lesions, patients will be evaluated in clinical follow-up visits at 1, 3, 6, and 9 months. In the final 9-month follow-up examination, patients will be evaluated by IVUS and VH to assess plaque volume and plaque composition in index non-PCI target intermediate lesions, and measure neointimal hyperplasia in index stented PCI target lesions. Lipid profiles, to include total cholesterol, LDL, HDL, triglyceride, apolipoprotein A-1 and B, LDL particle size and HDL subfractions, and biomarkers associated with atherosclerosis, including high-sensitivity C-reactive protein (hsCRP), oxidized LDL, VCAM-1, von Willebrand factor (vWF) and lipoprotein (a) [Lp(a)], will be measured at enrollment, and at 1- and 9-month follow-up examinations. The trial algorithm is shown in Figure 1. This is an investigator-initiated clinical trial with grant support from Korea Otska Pharmaceutical Co. Ltd (Seoul, Korea). Other than providing financial support, the company is not involved in developing study protocols or study processes, including site selection, management, and data collection and analysis. The study is performed according to the principles of the Declaration of Helsinki and according to common guidelines for clinical trials (ICH-GCP). The authors are solely responsible for the design and editing of this paper and its final content. The trial protocol has been approved by the local institutional review boards of each patient-enrolling center, and has been registered at http://www.clinicaltrials.gov (NCT01031667). Patient information, blood samples, and IVUS, VH and coronary angiogram images will be coded with the identification number of this study; investigators are blinded to patient identifying information, which has been replaced with a coded number.

### Study population and Randomisation

Patients more than 20 years of age who have had at least one significant coronary artery stenosis requiring PCI with stent implantation and one intermediate lesion on a native coronary artery with luminal narrowing of ≥ 30% and ≤ 70% by visual estimation, and who also meet all inclusion and exclusion criteria (Table 1) are eligible for this study. Those who agree to participate in the study and give written consent are randomised (1:1) to either cilostazol and probucol combination therapy or cilostazol monotherapy (control) using a web-based online randomization system. The randomization is stratified by enrolling site and the presence of diabetes in order to ensure an even distribution between the two groups of major factors that might contribute to the primary end point. After enrollment, all patients will receive a starting dose of 20 mg simvastatin. The dose of simvastatin will be adjusted to reach a target LDL level of < 100 mg/dL. Concomitant use of other drugs that interfere with serum lipid levels, such as niacin, CETP or lipoprotein-associated phospholipase A2 (Lp-PLA2) inhibitor, fenofibrate or omega-3, is not allowed.

**Table 1:** Patient Inclusion and Exclusion Criteria

Inclusion criteria
1. Male or female over 20 years of age
2. Clinical indication for coronary angiography
3. Presence of at least one significant coronary artery stenosis (>50%) requiring PCI with stent implantation and one intermediate lesion with luminal narrowing of ≥30% and <70% by visual estimation on native coronary arteries.
4. Presence of at least one PCI target lesion (reference diameter 2.5~4 mm, lesion length ≤26 mm) with >50% diameter stenosis that can be covered with a single Zotrolimus-eluting stent (Endeavor Sprint, Medtronic Vascular Inc, Santa Rosa, CA).
5. Able to understand and willing to provide written informed consent
Exclusion criteria
1. Intermediate lesions that might provide difficulty for IVUS evaluation because of following reasons: heavy calcification (>90° Arc), tortuous vessel with severe angulation, total occlusion, or bifurcation lesions
2. Previous PCI in the last 6 months
3. Previous coronary artery bypass graft
4. Patients with AMI undergoing primary PCI or rescue PCI after thrombolysis
5. Cardiogenic shock
6. Inability to take adequate antiplatelet therapy (aspirin and clopidogrel)
7. Thrombocytopenia (platelet count <70 × 10^9^/l)
8. Known hypersensitivity or contraindication to any of the following medications: Heparin, aspirin, clopidogrel, cilostazol, probucol, statin, contrast media
9. History of severe ventricular arrhythmia
10. Significant QTc prolongation (≥470 ms) on electrocardiogram
11. Heart failure (New York Heart Association class III/IV) or left ventricular ejection fraction ≤35%
12. Familial hypercholesterolemia
13. Uncontrolled hypertriglyceridemia (>400 mg/dL)
14. Chronic renal failure with serum creatinine level ≥2 mg/dL
15. Severe liver disease or transaminase level ≥3 times the upper limit of normal.
16. Pregnancy or women at age of childbearing potential

An additional file "Figure 1" contains figure 1.

### Index intermediate lesion for evaluating plaque volume

An intermediate lesion is defined as a lesion with a luminal narrowing ≥ 30% and ≤ 70% (estimated visually) that does not require percutaneous coronary intervention. One such lesion located in the proximal or mid segment of coronary arteries is selected by the operator as the index intermediate lesion. Intermediate lesions that might prove difficult to evaluate by IVUS, such as bifurcating lesions, heavily calcified lesions (> 90° arc), and those in tortuous vessels with severe angulation are avoided in the selection of an index lesion.

### Percutaneous coronary intervention

All patients should be on chronic aspirin (100-325 mg/day) and clopidogrel (75 mg/day) therapy for ≥ 5 days or receive aspirin (250 mg) and clopidogrel (300-600 mg) loading at least 12 hours before PCI. Unfractionated heparin will be administered according to local standards of care (target ACT 250 sec) at a dosage as per label instructions. The use of glycoprotein IIb/IIIa inhibitors will be left to the discretion of the operator. The criteria for an index PCI target lesion are a stenotic lesion with a stenosis diameter > 50% and lesion length ≤ 26 mm that can be covered by a single stent. All PCI target lesions, including additional lesions not established as index lesions, will be treated with Zotarolimus-eluting stents (Endeavor Sprint, Medtronic Vascular Inc, Santa Rosa, CA). After implantation of stents, aspirin (100 mg/day) and clopidogrel (75 mg/day) will be administered during the period of study.

### Intravascular Ultrasound and Virtual Histology

All IVUS imaging will be performed using a 20-MHz 2.9F, phased-array IVUS catheter (Eagle Eye, Volcano Therapeutics, Rancho Cordova, CA) after first administering nitroglycerin (200 mg). The IVUS catheter is placed distal to the target lesion, and then pulled back using a motorized pullback system at 0.5 cm/s. During pullback, grayscale IVUS is recorded and raw radiofrequency data are captured at the top of the R wave for reconstruction of the color-coded map by a VH-IVUS data recorder (Volcano Therapeutics). For evaluation of the index intermediate lesion, a segment at least 30 mm in length will be evaluated with IVUS and VH at baseline and 9-month follow-up. The most severe 10-mm segment centered on the minimal lumen area will be analyzed for plaque volume and plaque composition (fibrotic, fibrofatty, dense calcium, and necrotic core components). The index PCI target lesion will be evaluated for neointimal growth with grayscale IVUS after PCI and at 9-month follow-up. IVUS evaluation will include 5-mm segments proximal and distal to the stent. IVUS and VH images will be analyzed by an independent core laboratory (Cardiac Core Analysis Laboratory at Stanford University Medical Center, Stanford, CA).

### Laboratory assessment

Blood samples will be obtained after overnight fasting. Serum lipids, apolipoproteins, hs-CRP, Lp(a), oxidized LDL, VCAM-1, and vWF levels will be measured at a central clinical laboratory (Seoul Clinical Laboratories, Seoul, Korea). Serum total cholesterol, LDL, HDL, and triglyceride levels will be measured using standard enzymatic methods. Apolipoprotein A-1 and B concentrations will be measured by turbidimetric immunoassay methods. Serum oxidized LDL, VCAM-1, and vWF levels will be measured using enzyme-linked immunosorbent assays. Serum hsCRP and Lp(a) levels will be measured using latex-enhanced turbidimetric immunoassays. The particle-size distribution of LDL isolated by sequential flotation ultracentrifugation will be determined using a pore-gradient lipoprotein system (CBS Scientific, CA) at the National Research Laboratory for Clinical Nutrigenetics/Nutrigenomics, Yonsei University, Seoul, Korea, as previously described [26]. Circulating HDL subfractions HDL2 and HDL3 will be quantified after ultracentrifugation of serum at the Laboratory of Nutritional Medicine/Nutrigenomics, Sungshin Women's University, Seoul, Korea, as previously reported [27].

### Study end points

The primary end point is the change in the plaque volume of index intermediate lesions between baseline and 9-month follow-up.

The secondary end points include the following:

• Plaque composition change by VH

• Clinical outcomes (death, myocardial infarction, target vessel revascularization)

• Neointimal volume obstruction at index PCI target lesions

• Blood lipid composition (total cholesterol, LDL, LDL particle size, HDL, HDL subfractions, apolipoprotein A-1 and B)

• Biomarkers (hsCRP, VCAM-1, oxidized LDL, Lp(a), vWF)

### Statistical considerations

#### Sample size calculation

The calculation of the sample size was based on an in-equality design and a two-sample, two-sided test. Assuming a standard deviation of 8%, an α-level of 0.05 and a statistical power of 80%, 41 patients are required for each group (combination therapy, cilostazol monotherapy) to demonstrate a 5% difference in the mean plaque volume in a 10-mm segment with the greatest disease severity of the index intermediate lesion. Making allowance for a drop-out rate of 30% increases the number of patients required for each group to 59. The treatment difference and standard deviation assumptions are based on previous clinical and animal studies. Several IVUS studies have successfully demonstrated the effect of statins in reducing plaque volume. Yokoyama et al. [22] showed a 3.2% reduction in plaque volume in patients treated with atorvastatin (10 mg/day) for 6 months. The ASTEROID trial achieved about a 25% reduction in the normalized total atheroma volume using rosuvastatin (40 mg/day) for 24 months [28]. Recently, Tardif at al. [29] reported that a 12-month therapy with succinobucol, an analog of probucol, reduced the plaque volume of index lesions by 1.5%, whereas the control group showed no significant change in plaque volume. Notably, the results of an animal study evaluating the antiatherogenic effects of cilostazol and probucol showed that combination therapy was more effective in reducing the size of atherosclerotic lesions than was administration of either drug alone [18]. For this reason, we expect that cilostazol and probucol combination therapy will be more potent in reducing plaque volume than succinobucol, and hypothesize that the combination therapy could achieve a reduction in plaque volume 5% greater than that achieved by cilostazol monotherapy.

### Statistical analysis

All statistical analyses will be carried out by an independent organization, ADM Korea Inc. All primary and secondary end points will be analyzed on a modified intention-to-treat basis (all randomized subjects who receive at least one dose of study drug and have IVUS and VH measurements at baseline and at the study end point). The primary efficacy analysis will be performed using an analysis of covariance model, adjusting for baseline values. If the data are not distributed normally, a nonparametric analysis of covariance analysis will be used. All continuous secondary efficacy variables will be analyzed using similar methods. The composite end point of cardiovascular events will be analyzed by comparing Kaplan-Meier event rates using a log-rank test. Independent predictors of major adverse cardiac events (MACE) were determined using a multiple logistic regression analysis.

### Trial Organization

*Principal Investigator *Won-Heum Shim, Division of Cardiology, Severance Cardiovascular Hospital, Yonsei University Health System, Seoul, Korea

*Statistical analysis *ADM, Korea Inc., Seoul, Korea

*Core laboratory for IVUS and VH *Peter J. Fitzerald and Yasuhiro Honda, Cardiac Core Analysis Laboratory at Stanford University Medical Center, Stanford, CA, USA

*Core laboratory for biomarkers *Seoul Clinical Laboratories, Seoul, Korea

*Core laboratory for LDL subfractions *Jong Ho Lee, National Research Laboratory for Clinical Nutrigenetics/Nutrigenomics, Yonsei University, Seoul, Korea

*Core laboratory for LDL subfractions *Myoungsook Lee, Laboratory of Nutritional Medicine/Nutrigenomics, Sungshin Women's University, Seoul, Korea

*Data coordination and site management *Novotech Korea, Seoul, Korea

## Discussion

SECURE has been designed to test the hypothesis that combination therapy with cilostazol and probucol is more effective in reducing plaque volume than cilostazol monotherapy. The effectiveness of probucol against atherosclerosis has been demonstrated in previous clinical trials. In the Fukuoka Atherosclerosis Trial (FAST), the patient group treated with probucol (500 mg/day) showed significantly reduced intima media thickness in the common carotid artery compared to the control group [30]. In an IVUS substudy of the Canadian Antioxidant Restenosis Trial (CART-2), significant atherosclerosis regression was observed in patients treated with the probucol analog succinobucol, whereas no significant change in plaque volume occurred in the placebo group [29]. In the Probucol Observational Study Illuminating Therapeutic Impact on Vascular Events (POSITIVE), long-term probucol treatment effectively prevented secondary cardiovascular events in a higher-cardiovascular-risk heterozygous familial hypercholesterolemia population without causing severe adverse effects [31]. Cilostazol has also demonstrated diverse antiatherogenic properties in previous studies [11-14]. Recent animal studies have suggested that probucol and cilostazol may synergize in the prevention of atherosclerosis by suppressing inflammatory reaction and promoting cholesterol efflux [18,19].

In the SECURE study, in addition to changes in plaque volume, we will also evaluate the potential of combination therapy to stabilize coronary plaques by altering their composition. Virtual Histology, a software system that enables a spectral analysis of radiofrequency backscatter IVUS, was recently developed to analyze plaque composition, providing information on fibrous, fibrofatty, dense calcium, and necrotic core components [20]. VH findings have been validated using various human atherosclerotic plaque tissues [21]. Different statins and darapladip, a direct lipoprotein-associated phospholipase A(2) inhibitor, have shown coronary plaque-stabilizing effects by reducing the necrotic core [22-25]. The SECURE study will also confirm whether the combination therapy is superior to cilostazol alone in decreasing neointimal hyperplasia after implantation of a drug-eluting stent. Previous studies have shown that both probucol and cilostazol are effective in preventing restenosis after PCI with or without stent implantation [2,10,32]. In addition, Sekiya et al. [17] demonstrated that cilostazol and probucol combination therapy reduced restenosis after stent implantation more effectively than either cilostazol or probucol alone.

Another focus of the SECURE study is on changes in lipid composition associated with probucol and cilostazol combination therapy. Generally, high LDL and low HDL cholesterol levels are regarded as major risk factors for coronary artery disease. However, within LDL and HDL cholesterols are subfractions with different particle sizes and different roles in the metabolism of lipids [33,34]. Although still controversial, small, dense LDL is thought to be more atherogenic in conjunction with other features of metabolic syndrome, such low HDL and high triglyceride levels [33]. Generally, the larger triglyceride-rich HDL2 subfraction is thought to confer better protection against coronary artery disease than the small HDL3 fraction [34]. In a recent trial, the CETP inhibitor torcetrapib, which raised HDL-C levels by approximately 70%, showed a paradoxical increase in cardiovascular disease outcomes [35]. Thus, a simple elevation of HDL cholesterol levels does not necessarily translated into protection from atherosclerosis. Probucol is thought to reduce HDL cholesterol by enhancing reverse cholesterol transport through increased expression of the hepatic HDL-receptor SR-B1 and activation of CETP [4-6]. Hepatic over-expression of SR-B1 increases hepatic uptake of HDL cholesterol resulting in increased biliary excretion of cholesterol. Probucol is known to increase the levels and activity of CETP [4,5]. Activation of CETP increases transfer of cholesterol esters from HDL to triglyceride-rich lipoprotein particles in exchange for triglyceride, thereby reducing the circulating HDL cholesterol concentration. Whether CETP is anti- or proatherogenic is currently not fully understood. Potential antiatherogenic roles of CETP include HDL remodeling, with increased efflux of lipid-poor apolipoprotein A-I in exchange for cholesterol; and shuttling of cholesteryl esters to apolipoprotein B-containing lipoproteins, namely LDL, intermediate density lipoprotein (IDL), and very low density lipoprotein (VLDL), for excretion by the liver. In the SECURE study, we will investigate lipid parameters, such as LDL and HDL subfractions, and apolipoprotein A1 and B, as well as total cholesterol, triglyceride, HDL, and LDL. In addition, we will evaluate the effects of probucol and cilostazol combination therapy on the atherosclerosis biomarkers, oxidized LDL, VCAM-1, and vWF.

Possible major limitations of the SECURE study will be the relatively small number of study population and the short interval duration for follow-up IVUS study. However, several small clinical studies have shown significant plaque reduction with change of plaque composition despite relatively short period (6 or 12 months) of follow-up [22,23,25]. Thus, if probucol combined with cilostazol has strong antiatherosclerotic effects as shown in previous clinical and experimental studies, we think the SECURE study will be able to demonstrate them in terms of the primary or secondary endpoints.

In conclusion, the SECURE study will be the first double-blind, randomized, controlled multicenter clinical trial using VH-IVUS to investigate the effects of probucol and cilostazol combination therapy on the progression and composition of coronary artery plaques. Furthermore, the SECURE study will deliver important information on the effects of combination therapy on lipid composition and biomarkers related to atherosclerosis, thereby providing insight into the mechanisms underlying the prevention of atherosclerosis progression by cilostazol and probucol.

## Trial registration number

National Institutes of Health Clinical Trials Registry (ClinicalTrials.gov identifier #NCT01031667).

## Abbreviations

LDL: low-density lipoprotein; CAD: coronary artery disease; PCI: percutaneous coronary intervention; HDL: high density lipoprotein; CETP: cholesteryl ester transfer protein; SR-B1: scavenger reverse cholesterol class B type 1; VCAM: vascular cell adhesion molecule; MCP: monocyte chemoattractant protein; IVUS: intravascular ultrasound; VH: Virtual Histology; hsCRP: high-sensitivity C-reactive protein; vWF: von Willebrand factor; Lp(a): lipoprotein (a); Lp-PLA2: lipoprotein-associated phospholipase A2; MACE: major adverse cardiac events

## Competing interests

All authors declare that they have no competing interests and did not receive any honorarium from Korea Otska Pharmaceutical Co. Ltd or other partners. The investigator-initiated grant received by Korea Otska Pharmaceutical Co. Ltd guarantees independent conceivability of the study design, its coordination, realization and independent report of the study results.

## Authors' contributions

YK and BK participated in the conception and design of the study and contributed equally to the preparation of this manuscript. BL, WK, SC, and SK will enroll patients and collect clinical and IVUS data. JL and ML will analyze HDL and LDL subtypes. P F and YH will analyze IVUS and VH images in a core lab. SW is the principal investigator and initiator of the study, obtained funding, designed the study and supervised and participated in writing the manuscript. All authors read, and approved the final manuscript.

## References

[B1] Baigent C Keech A Kearney PM Blackwell L Buck G Pollicino C Kirby A Sourjina T Peto R Collins R Simes R Cholesterol Treatment Trialists' (CTT) Collaborators Efficacy and safety of cholesterol-lowering treatment: prospective meta-analysis of data from 90,056 participants in 14 randomized trials of statins Lancet 2005 366 1267 1278 10.1016/S0140-6736(05)67394-1 16214597

[B2] Yamashita S Matsuzawa Y Where are we with probucol: a new life for an old drug? Atherosclerosis 2009 207 16 23 10.1016/j.atherosclerosis.2009.04.002 19457483

[B3] Sasahara M Raines EW Chait A Carew TE Steinberg D Wahl PW Ross R Inhibition of hypercholesterolemia induced atherosclerosis in the nonhuman primate by probucol. I. Is the extent of atherosclerosis related to resistance of LDL to oxidation? J Clin Invest 1994 94 155 164 10.1172/JCI117301 8040256 PMC296293

[B4] Franceschini G Sirtori M Vaccarino V Gianfranceschi G Rezzonico L Chiesa G Sirtori CR Mechanisms of HDL reduction after probucol. Changes in HDL subfractions and increased reverse cholesteryl ester transfer Arteriosclerosis 1989 9 462 469 2751476 10.1161/01.atv.9.4.462

[B5] Ishigami M Yamashita S Sakai N Hirano K Arai T Maruyama T Takami S Koyama M Kameda-Takemura K Matsuzawa Y High-density lipoproteins from probucol-treated patients have increased capacity to promote cholesterol efflux frommouse peritoneal macrophages loaded with acetylated low-density lipoproteins Eur J Clin Invest 1997 27 285 292 10.1046/j.1365-2362.1997.1040657.x 9134376

[B6] Hirano K Ikegami C Tsujii K Zhang Z Matsuura F Nakagawa-Toyama Y Koseki M Masuda D Maruyama T Shimomura I Ueda Y Yamashita S Probucol enhances the expression of human hepatic scavenger receptor class B type I, possibly through a species-specific mechanism Arterioscler Thromb Vasc Biol 2005 25 2422 2427 10.1161/01.ATV.0000185834.98941.3d 16151015

[B7] Miida T Seino U Miyazaki O Hanyu O Hirayama S Saito T Ishikawa Y Akamatsu S Nakano T Nakajima K Okazaki M Okada M Probucol markedly reduces HDL phospholipids and elevated prebeta1-HDL without delayed conversion into alpha-migrating HDL: Putative role of angiopoietin-like protein 3 in probucolinduced HDL remodeling Atherosclerosis 2008 200 329 335 10.1016/j.atherosclerosis.2007.12.031 18279878

[B8] Fruebis J Gonzalez V Silvestre M Palinski W Effect of probucol treatment on gene expression of VCAM-1, MCP-1, and M-CSF in the aortic wall of LDL receptor-deficient rabbits during early atherogenesis Arterioscler Thromb Vasc Biol 1997 17 1289 1302 9261259 10.1161/01.atv.17.7.1289

[B9] Lau AK Leichtweis SB Hume P Mashima R Hou JY Chaufour X Wilkinson B Hunt NH Celermajer DS Stocker R Probucol promotes functional reendothelialization in balloon-injured rabbit aortas Circulation 2003 107 2031 2036 10.1161/01.CIR.0000062682.40051.43 12681995

[B10] Weintraub WS The vascular effects of cilostazol Can J Cardiol 2006 22 Suppl B 56B 60B 16498513 10.1016/s0828-282x(06)70987-4 PMC2780845

[B11] Nakamura T Houchi H Minami A Sakamoto S Tsuchiya K Niwa Y Minakuchi K Nakaya Y Endothelium-dependent relaxation by cilostazol, a phosphodiesteras III inhibitor, on rat thoracic aorta Life Sci 2001 69 1709 1715 10.1016/S0024-3205(01)01258-9 11665832

[B12] Kim KY Shin HK Choi JM Hong KW Inhibition of lipopolysaccharide-induced apoptosis by cilostazol in human umbilical vein endothelial cells J Pharmacol Exp Ther 2002 300 709 715 10.1124/jpet.300.2.709 11805237

[B13] Okutsu R Yoshikawa T Nagasawa M Hirose Y Takase H Mitani K Okada K Miyakoda G Yabuuchi Y Cilostazol inhibits modified low-density lipoprotein uptake and foam cell formation in mouse peritoneal macrophages Atherosclerosis 2009 204 405 411 10.1016/j.atherosclerosis.2008.10.042 19108834

[B14] Ishizaka N Taguchi J Kimura Y Ikari Y Aizawa T Togo M Miki K Kurokawa K Ohno M Effects of a single local administration of cilostazol on neointimal formation in balloon-injured rat carotid artery Atherosclerosis 1999 142 41 46 10.1016/S0021-9150(98)00147-6 9920504

[B15] Tani T Uehara K Sudo T Marukawa K Yasuda Y Kimura Y Cilostazol, a selective type III phosphodiesterase inhibitor, decreases triglyceride and increases HDL cholesterol levels by increasing lipoprotein lipase activity in rats Atherosclerosis 2000 152 299 305 10.1016/S0021-9150(99)00480-3 10998457

[B16] Elam MB Heckman J Crouse JR Hunninghake DB Herd JA Davidson M Gordon IL Bortey EB Forbes WP Effect of the novel antiplatelet agent cilostazol on plasma lipoproteins in patients with intermittent claudication Arterioscler Thromb Vasc Biol 1998 18 1942 1947 9848888 10.1161/01.atv.18.12.1942

[B17] Sekiya M Funada J Watanabe K Miyagawa M Akutsu H Effects of probucol and cilostazol alone and in combination on the frequency of poststenting restenosis Am J Cardiol 1998 82 144 147 10.1016/S0002-9149(98)00323-3 9678282

[B18] Yoshikawa T Mitani K Kotosai K Nozako M Miyakoda G Yabuuchi Y Antiatherogenic effects of cilostazol and probucol alone, and in combination in low density lipoprotein receptor-deficient mice fed with a high fat diet Horm Metab Res 2008 40 473 478 10.1055/s-2008-1065348 18404599

[B19] Park SY Lee JH Shin HK Kim CD Lee WS Rhim BY Shin YW Hong KW Synergistic efficacy of concurrent treatment with cilostazol and probucol on the suppression of reactive oxygen species and inflammatory markers in cultured human coronary artery endothelial cells Korean J Physiol Pharmacol 2008 12 165 170 10.4196/kjpp.2008.12.4.165 19967051 PMC2788631

[B20] Nair A Kuban BD Tuzcu EM Schoenhagen P Nissen SE Vince DG Coronary plaque classification with intravascular ultrasound radiofrequency data analysis Circulation 2002 106 2200 2206 10.1161/01.CIR.0000035654.18341.5E 12390948

[B21] Nasu K Tsuchikane E Katoh O Vince DG Virmani R Surmely JF Murata A Takeda Y Ito T Ehara M Matsubara T Terashima M Suzuki T Accuracy of in vivo coronary plaque morphology assessment: a validation study of in vivo virtual histology compared with in vitro histopathology J Am Coll Cardiol 2006 47 2405 2412 10.1016/j.jacc.2006.02.044 16781367

[B22] Yokoyama M Komiyama N Courtney BK Nakayama T Namikawa S Kuriyama N Koizumi T Nameki M Fitzgerald PJ Komuro I Plasma low-density lipoprotein reduction and structural effects on coronary atherosclerotic plaques by atorvastatin as clinically assessed with intravascular ultrasound radio-frequency signal analysis: A randomized prospective study. the effects of drugs on stabilization of coronary atherosclerotic plaques Am Heart J 2005 150 287.e1 287.e7 10.1016/j.ahj.2005.03.059 16086932

[B23] Nasu K Tsuchikane E Katoh O Tanaka N Kimura M Ehara M Kinoshita Y Matsubara T Matsuo H Asakura K Asakura Y Terashima M Takayama T Honye J Hirayama A Saito S Suzuki T Effect of fluvastatin on progression of coronary atherosclerotic plaque evaluated by virtual histology intravascular ultrasound JACC Cardiovasc Interv 2009 2 689 696 10.1016/j.jcin.2009.04.016 19628194

[B24] Serruys PW García-García HM Buszman P Erne P Verheye S Aschermann M Duckers H Bleie O Dudek D Bøtker HE von Birgelen C D'Amico D Hutchinson T Zambanini A Mastik F van Es GA van der Steen AF Vince DG Ganz P Hamm CW Wijns W Zalewski A Integrated Biomarker and Imaging Study-2 Investigators Effects of the direct lipoprotein-associated phospholipase A(2) inhibitor darapladib on human coronary atherosclerotic plaque Circulation 2008 118 1172 1182 10.1161/CIRCULATIONAHA.108.771899 18765397

[B25] Hong MK Park DW Lee CW Lee SW Kim YH Kang DH Song JK Kim JJ Park SW Park SJ Effects of statin treatments on coronary plaques assessed by volumetric virtual histology intravascular ultrasound analysis JACC Cardiovasc Interv 2009 2 679 688 10.1016/j.jcin.2009.03.015 19628193

[B26] Jang Y Paik JK Hyun YJ Chae JS Kim JY Choi JR Lee SH Shin DJ Ordovas JM Lee JH The apolipoprotein A5-1131T>C promoter polymorphism in Koreans: association with plasma APOA5 and serum triglyceride concentrations, LDL particle size and coronary artery disease Clin Chim Acta 2009 402 83 87 10.1016/j.cca.2008.12.024 19159622 PMC4428346

[B27] Patsch W Brown SA Morrisett JD Gotto AM Jr Patsch JR A dual-precipitation method evaluated for measurement of cholesterol in high-density lipoprotein subfractions HDL2 and HDL3 in human plasma Clin Chem 1989 2 265 270 2914371

[B28] Nissen SE Nicholls SJ Sipahi I Libby P Raichlen JS Ballantyne CM Davignon J Erbel R Fruchart JC Tardif JC Schoenhagen P Crowe T Cain V Wolski K Goormastic M Tuzcu EM ASTEROID Investigators Effect of very high-intensity statin therapy on regression of coronary atherosclerosis. The ASTEROID Trial JAMA 2006 295 1556 1565 10.1001/jama.295.13.jpc60002 16533939

[B29] Tardif JC Grégoire J L'Allier PL Ibrahim R Anderson TJ Reeves F Title LM Schampaert E LeMay M Lespérance J Scott R Guertin MC Brennan ML Hazen SL Bertrand OF; CART-2 Investigators Effects of the antioxidant succinobucol (AGI-1067) on human atherosclerosis in a randomized clinical trial Atherosclerosis 2008 197 480 486 10.1016/j.atherosclerosis.2006.11.039 17214993

[B30] Sawayama Y Shimizu C Maeda N Tatsukawa M Kinukawa N Koyanagi S Kashiwagi S Hayashi J Effects of probucol and pravastatin on common carotid atherosclerosis in patients with asymptomatic hypercholesterolemia. Fukuoka Atherosclerosis Trial (FAST) J Am Coll Cardiol 2002 39 610 616 10.1016/S0735-1097(01)01783-1 11849859

[B31] Yamashita S Bujo H Arai H Harada-Shiba M Matsui S Fukushima M Saito Y Kita T Matsuzawa Y Long-term probucol treatment prevents secondary cardiovascular events: a cohort study of patients with heterozygous familial hypercholesterolemia in Japan J Atheroscler Thromb 2008 15 292 303 19060422 10.5551/jat.e610

[B32] Tardif JC Cöté G Lespérance J Bourassa M Lambert J Doucet S Bilodeau L Nattel S de Guise P Probucol and multivitamins in the prevention of restenosis after coronary angioplasty. Multivitamins and Probucol Study Group N Engl J Med 1997 337 365 372 10.1056/NEJM199708073370601 9241125

[B33] Superko HR Gadesam RR Is it LDL particle size or number that correlates with risk for cardiovascular disease? Curr Atheroscler Rep 2008 10 377 385 10.1007/s11883-008-0059-2 18706278

[B34] Colvin PL Parks JS Metabolism of high density lipoprotein subfractions Curr Opin Lipidol 1999 10 309 314 10.1097/00041433-199908000-00004 10482133

[B35] Tall AR Yvan-Charvet L Wang N The Failure of Torcetrapib: was it the Molecule or the Mechanism? Arterioscler Thromb Vasc Biol 2007 27 257 260 10.1161/01.ATV.0000256728.60226.77 17229967

